# GEOGLE: context mining tool for the correlation between gene expression and the phenotypic distinction

**DOI:** 10.1186/1471-2105-10-264

**Published:** 2009-08-25

**Authors:** Yao Yu, Kang Tu, Siyuan Zheng, Yun Li, Guohui Ding, Jie Ping, Pei Hao, Yixue Li

**Affiliations:** 1 Key Lab of Systems Biology, Shanghai Institutes for Biological Sciences, Chinese Academy of Sciences, Shanghai 200031, PR China; 2 Graduate School of the Chinese Academy of Sciences, Shanghai 200031, PR China; 3 Shanghai Center for Bioinformation Technology, 100 Qinzhou Road, Shanghai 200235, PR China; 4 College of life science and biotechnology, Shanghai Jiaotong University, Shanghai 200240, PR China; 5 College of life science and biotechnology, Shanghai Tongji University, Shanghai 200331, PR China

## Abstract

**Background:**

In the post-genomic era, the development of high-throughput gene expression detection technology provides huge amounts of experimental data, which challenges the traditional pipelines for data processing and analyzing in scientific researches.

**Results:**

In our work, we integrated gene expression information from Gene Expression Omnibus (GEO), biomedical ontology from Medical Subject Headings (MeSH) and signaling pathway knowledge from sigPathway entries to develop a context mining tool for gene expression analysis – GEOGLE. GEOGLE offers a rapid and convenient way for searching relevant experimental datasets, pathways and biological terms according to multiple types of queries: including biomedical vocabularies, GDS IDs, gene IDs, pathway names and signature list. Moreover, GEOGLE summarizes the signature genes from a subset of GDSes and estimates the correlation between gene expression and the phenotypic distinction with an integrated p value.

**Conclusion:**

This approach performing global searching of expression data may expand the traditional way of collecting heterogeneous gene expression experiment data. GEOGLE is a novel tool that provides researchers a quantitative way to understand the correlation between gene expression and phenotypic distinction through meta-analysis of gene expression datasets from different experiments, as well as the biological meaning behind. The web site and user guide of GEOGLE are available at:

## Background

The rapid development of high-throughput gene expression detection technology provides a huge amount of experimental data for advanced researches on associating gene expression signatures with biological phenotypes. The application of microarrays to identify gene expression signatures of human diseases has been widely accepted [1,2]. Accordingly, a vast number of tools for microarray analysis are available, including ArrayPipe [3], GEPAS [4], GeneTrailExpress [5], and currently reported Perl modules for microarray analysis [6], etc. Besides, Gene Set Analysis is highlighted in microarray analysis. Gene sets are usually defined as set of genes which function in cohort, detailed analysis on which can lead to a functional level map of the transcriptome data. Some popular gene set analysis tools include Babelomics [7], WebGestalt [8], etc. Furthermore, to address the problems of limited samples in single biological experiment and heterogeneity of gene expression datasets from different sources, methods for large-scale meta-analysis of microarray data have been developed [9-11]. Those tools for meta-analysis like studies such as Connectivity Map [12] requires a huge amount of supporting data resources, and associated information from existing biological databases. There is a clear requirement for efficiently retrieving associative datasets for meta-analysis to avoid manual work in mining from a large number of references.

The Gene Expression Omnibus (GEO) [13], curated by the National Center for Biotechnology Information (NCBI), is designed in response to this demand as a public warehouse for the submission, storage and retrieval of the high-throughput gene expression and genomic hybridization experiments. Several tools and strategies for operating the GEO database have been developed to enable comparisons of microarray data across experimental platforms, different laboratories and multiple species [14-18]. However most of these tools for retrieving data from the GEO repository paid little attention to mining further information about the gene expression signatures, such as linking to the biological functions of genes, or integrating the related pathway information in the biological processes. The National Library of Medicine's controlled vocabulary thesaurus (MeSH) [19] is one of the best resources for biomedical vocabularies. MeSH is helpful to be used as an index to link experimental conditions and biological concepts together, including disease phenotypes.

Focusing on this issue, we developed a state-of-the-art online bioinformatics tool, named GEOGLE, for mining the experimental data from GEO database and constructing relationships among the datasets, genes, pathways and the genes' biological significance. Our system integrates information from multiple sources, such as sigPathway [20] for pathway information and MeSH for biomedical vocabularies. Investigators are able to use multiple types of data for querying – including disease information, gene symbols, pathway names, expression datasets (GDS IDs), and signature lists – to search a large collection of related microarray information. An integrated p value is introduced by GEOGLE, which could be considered as an estimate for the correlation between gene expression and the phenotypic distinction. This mining technology may have great value in discovering the linkages between known phenotypes and experiment data, as well as retrieving suitable datasets for further research work.

## Implementation

### GEOGLE description and results

The analysis in GEOGLE consists of two major parts: meta-analysis which integrates literature information and similarity search for signatures and datasets. The gene expression data and the basic signature for each dataset are derived from public expression data warehouses, such as GEO. MeSH terms have been used as important vocabulary dictionary for associating gene expression data with other biological terms, such as pathways and diseases. Dataset searching is mainly based on checking synonyms from pathways or diseases' description of their relevant MeSH terms in dataset annotation. Through literature searching and dataset filtering, summarized signatures are available from the integration of GEO, MeSH and sigPathway. For the second part of signature similarity search, a similar method has been used in Connectivity Map [12]. GEOGLE will search similar datasets sharing the same signatures from the databases. By associating the attributes of these datasets, GEOGLE will summarize the common features and suggest the potential relationships between genes and diseases.

### Datasets collection and signature extraction

**Figure 1:**
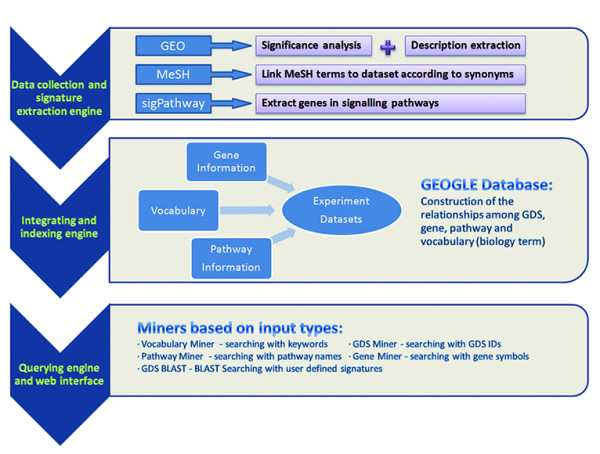
**The components of GEOGLE**. GEOGLE contains three major engines: the data collection and signature extraction engine, the integrating and indexing engine, the querying engine and web interface.

An architectural pattern was used in the design of GEOGLE that isolated data processing logic from user interface considerations. Each part of this pattern is independent for more convenient maintenance (Fig. 1). The first module is a data collection and signature extraction engine. Currently, gene expression datasets collected in GEOGLE mainly consist of data in three species from GEO: Human, Mouse and Rat. In total 1005 GEO datasets (GDSes) from 21 platforms (GPLs) have been collected in GEOGLE. 6 GPLs in Human contain 351 GDSes, while 9 GPLs in Mouse contain 500 GDSes and 6 GPLs in Rat contain 154 GDSes. The details of these datasets are available from our web site. Some GEO datasets will be filtered out because of the limited chip numbers (i.e. less than 3 samples in each subset), which might be considered not suitable for meta-analysis. Each dataset derived was classified into different groups according to corresponding experimental factors (e.g. tissue, strain, time, dose, etc). For example, GDS1436 (Cigarette smoking effect on alveolar macrophages) contains 10 samples. According to GSMs' labels these samples can be classified into two groups: non-smoker and smoker group. After classification, differential expression of each gene in each data set is tested using Significance Analysis of Microarray (SAM). SAM is a statistical technique for finding differentially expressed genes in microarray experiments . The p values from SAM reflect the correlation between gene expression and the phenotypic distinction. (A detail description of SAM should be found in the supplementary file) Biomedical vocabulary information was collected from MeSH. Considering the hierarchical structure of MeSH, we automatically associated the corresponding MeSH terms to all its stored synonyms. This engine can be also used to update GEOGLE by collecting data from web sources.

### Data integration and indexing

These data are processed by a second integrating and indexing engine. Three kinds of relationships were constructed through this engine and stored in the GEOGLE database, such as the linkage between gene and experiment dataset, between pathway and dataset, and between vocabulary and dataset. The linkage between gene and experiment dataset was represented in two aspects: the individual p value for estimating the significance of differential gene expression in one dataset, and the integrated p value for estimating the correlation between gene expression and the phenotypic distinction which might contain several datasets with similar phenotypes. The algorithm for calculating the integrated p value is presented in the next paragraph. To construct the linkage between pathway and dataset, signatures from datasets were mapped into pathways based on information from sigPathway dataset. MeSH terms were mapped into experiments' annotation according to the context mining of the recorded synonyms from the description of GDSes.

The procedure for calculating the integrated p value consists of two major parts. Firstly, the p value of each gene in individual dataset was calculated with SAM method, as mentioned before. Secondly, a novel procedure was developed to calculate an integrated p value for evaluating the relationship between signature and a group of referred datasets (reflecting a phenotype). Steps from (1) to (5) were performed:

(1) The p values of different genes in each dataset were organized into a vector. Then gene – GDS matrices (named *P*_*gc*_, *gc *for gene – condition) were generated from a set of p value vectors calculated independently from different GDSes. Each element in *P*_*gc *_represents a p value which had been prepared before using SAM.

(2) What we want to know is if these genes in *P*_*gc *_are perturbed under a group of GDSes, which equal to test if the sub matrix of *P*_*gc *_of those genes and those GDSes (named *P*_*gc*_*sub*_) follow uniform distribution. To perform such a test, *P*_*gc*_*sub *_was transformed to *Z*_*gc*_*sub *_with quantile function of normal distribution. A quantile function of a probability distribution is the inverse *F*^-1 ^of its cumulative distribution function (cdf) *F*. Assuming a continuous and strictly monotonic distribution function, *F:R *-> (0,1), the quantile function returns the value below which random draws from the given distribution would fall, *p *× 100 percent of the time. That is, it returns the value of x such that



The cumulative distribution function (cdf) of the normal distribution is expressed in terms of the density function as follows:



(3) Then *Z *score was summarized from *Z*_*gc*_*sub *_with the function:

, *n *for the number of elements in *Z*_*gc*_*sub*_

(4) A new p value was calculated to represent the significance of *Z *score using the cumulative distribution function of normal distribution (as mentioned in (2)). Let a parameter ('alpha') be the threshold of the p value from this test.

(5) If these genes were not signatures of a group of GDSes, the *P*_*gc*_*sub *_would follow uniform distribution. If *P*_*gc*_*sub *_followed uniform distribution, *Z*_*gc*_*sub *_would follow norm distribution. As a result, Z score would also follow norm distribution. A significant small value of Z comparing to normal distribution corresponded to the significantly being perturbed of these genes under these conditions. The p value from this test is considered as the integrated p value from the whole searching task. We could judge whether certain gene should be considered as signature in the group of GDSes by the integrated p value.

(6) To judging the relationship between candidate signatures and vocabularies is very similar with the procedure from (1) to (5) mentioned before. Each vocabulary (MeSH term) contains a groups of expression datasets (GDSes). The integrated p value for the significance of the correlation between signature and certain vocabulary is equal to that for the correlation between signature and the GDSes in the vocabulary.

(7) The next step is to evaluate the relationship between pathway and a group of genes (signatures). We used a very similar procedure with some modification. We constructed a pathway – gene matrices (named *P*_*pg*_, *pg *for pathway – gene). The relationship of pathways and genes were derived from sigPathway. Each element in this matrix is the integrated p value of gene in a group of GDSes (this group is determined according pervious GDS searching). Then the procedure from (1) to (5) was repeated, using *P*_*pg *_taking the place of *P*_*gc *_as initial input. The new integrated p values calculated were considered to be the estimate of the significance of the pathways in the searching task.

## Results

**Figure 2:**
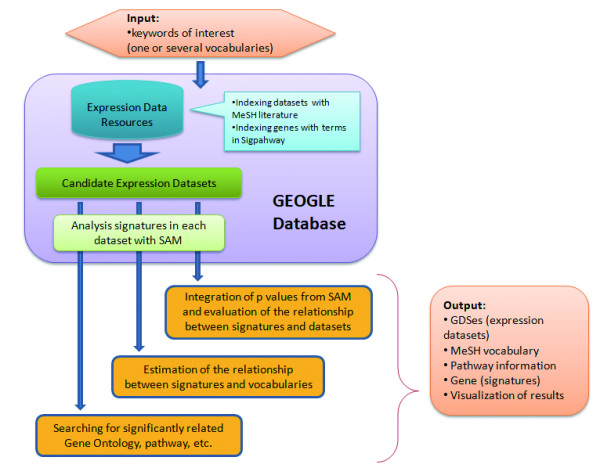
**The procedure for Vocabulary Miner**. This figure illustrates the main process in performing Vocabulary Miner. User can input certain keyword(s) for searching. GOEGLE will give the results contain: GDSes (expression datasets), significantly related MeSH vocabulary, involved pathway information, genes (signatures) and some visualization of results.

The third part of the pattern is a querying engine where users can perform searching tasks with a friendly web interface. The implementation of this engine is based on workflow technique using R and Java for script language and Omics Explorer (manuscript under review) as a web container. There are five miners provided by GEOGLE. The main difference of these miners includes various input types for querying including biomedical vocabularies, GDS identifies, pathway names, gene identifies or a set of signatures, and slightly different searching strategies. **Vocabulary Miner **(Fig. 2): Mining out relevant GDSes, common signature genes and pathways based on biomedical vocabularies. The submitted keywords (one or several vocabularies) of users' interest will be mapped to a series of related GDS according to MeSH dictionary and the description of GDS. The integrated P value which describes the relationship between each gene and the group of returned GDSes (as well as MeSH terms) is calculated. Meanwhile, the P value which describes the relationship between each pathway and the group of GDSes is calculated. Then significantly associated GDSes, genes and pathways will be individually sorted based on the p values and be given out with necessary annotation. **GDS Miner**: This miner is similar with the first one. However the datasets (usually GDS IDs) should be directly given by the users instead of being found by searching keywords. The following process of retrieving signature genes and extracting related pathway information is the same as Vocabulary Miner. Also this miner can be helpful in searching for the annotation information of datasets with GDS IDs. **Pathway Miner**: Users may submit certain pathway name or partial name. This miner will help to obtain those genes within the pathway. Then the GDSes in which these genes are considered as signatures will be returned as well as their annotation. Similarly, GEOGLE will summarize the correlation between pathways (or genes in the pathways) and different datasets. **Gene Miner**: Users can provide several genes of their interest directly as input. Then the miner will search for the associative GDSes in which these genes are considered to be signatures. The remaining process is quite similar with Pathway Miner. **GDS Blast Miner**: Users may submit a list of signatures according to their own definition. What this miner will perform is to search for similar datasets containing the same (or similar) group of signatures. Then this miner will summarize associated biomedical vocabularies to these GDSes. The format of submitted datasets is very flexible, examples of which could be a gene list with expression values or probe set IDs with FDR values (see Additional file 1).

Some optional parameters can be set by users. For instance, in Vocabulary Miner setting 'F' (false) for 'list_Mesh_GDS.only' makes the miner search additional information about genes and pathways according to the query. A more efficient searching, by setting 'T' for this parameter, comes at the cost of performing no pathway and gene information searching. Another common parameter is 'alpha'. This parameter sets the threshold for the integrated p value to estimate the correlation between gene expression and experiment datasets. GEOGLE provides a task management system for users to review the states of their previously conducting tasks and to retrieve the results later, which will be saved temporally on the server. The detail processing pipelines of these miners and a step-by-step tutorial of using GEOGLE and the explanation of the input and results could be found in the Supplementary.

By using 'Vocabulary Miner', we search for 'smoking' related gene expression gene data in human, then got a result with 4 GDSes considered to be candidate datasets (GDS1304, GDS1436, GDS1673 and GDS534). According to their annotation, these GDSes which are all related to cigarette smoking effect are suitable for further meta-analysis. Terms like 'Breast Cancer/Estrogen Receptor Signaling' and 'Stress Response to Cellular Damage' are returned with significant p values in pathway section of the results, which suggests that these pathways are closely related to the 'smoking' phenotype. Such genes like GALNT1 are identified as signature genes trough all these dataset. According to pervious report, GALNT1 is strongly associated with the using of tobacco and the risk of lung cancer [21,22]. In our previous work, GEOGLE severed as main tool for expression data analysis associated with metabolomics data, which reveals distinct variations related to nicotine consumption in human [23]. The combination of several miners provided not only suitable expression datasets but also candidate genes which might be related to the influence of smoking. The gene for alkylglycerone phosphate synthase (*alkyl-DHAP*, or *AGPS*) has been found strong down-regulated in smokers in human lung tissues. This is consistent with metabolic profiling. The down-regulation of this gene was found to influence both ether lipid and glycerophospholipid pathways, and shift the ratios of plasmalogens to diacyl-phosphatidylcolines.

## Conclusion

In this report we introduce GEOGLE, an online web service for GEO dataset mining and biomedical information integration. GEOGLE provides an efficient way for users to search for related experiment datasets according to their own research interest with various types of input. Another significant feature of GEOGLE is the novel concept of an integrating system for signatures, pathways, biological terms and disease information. Public data warehouses such as GEO are high-quality resources for an automatic mining and integration system of gene expression datasets and reference literatures from GEOGLE, which will be a revolution compared to manually collecting experimental data for biological research. Currently there exist a few tools for operating the GEO database. For example, Oncomine [24,25] is a previously published cancer gene expression analysis platform. CleanEx [26] also contains re-annotate experiment datasets with the MeSH terms and some on-line analysis tools for gene expression data. Compared with these tools, GEOGLE has some outstanding features and additional values for this kind of study. Firstly, the main object of GEOGLE is to search for candidate datasets from different experiments for further meta-analysis, according to certain biological vocabularies and/or genes of interest. Secondly, GEOGLE provides a quantitative method to evaluate the correlation between each gene and a series of gene expression datasets which might represents certain phenotypic distinction. Thirdly, GEOGLE collected a wide range of information about different kinds of diseases including cancer (over 60,000 MeSH terms have been involved). Fourthly, GEOGLE performed further mining for related gene function information, pathway annotation and reference knowledge and introduced an integrated p value for estimating the correlation between gene expression and the phenotypic distinction. Fifthly, GEOGLE allows multiple types of inputs such as keywords, datasets, pathways, genes and user defined signatures. Technically, a modular design concept allows each part of GEOGLE to be replaced by a more advanced one, for instance another BLAST engine with more accuracy could be used for the similarity search. The container of GEOGLE (Omics Explorer) is hosted via a standard online service platform supported by InforSense Ltd. Thus no individual GUI will be need for GEOGLE's online user interface. In addition, the GEOGLE database can be easily updated to keep it synchronized with public gene expression databases.

Further steps in the development of GEOGLE should focus on the integration of high-throughput gene expression databases other than GEO, such as the ArrayExpress [27] and the Stanford Microarray Database (SMD) [28]. One of the improvements of GEOGLE in-progress is large scale gene and disease information mining effort from reference databases [29] and integrating this information with existing signature data. The reference mining results are believed to be able to prove the reliability of the relationships between signatures and diseases discovered by GEOGLE. Moreover, since GEOGLE provides a potential network of diseases, genes and pathways, more analysis work focusing on this will be considered in future.

## Availability and requirements

• **Project name**: GEOGLE

• **Project home page**: 

• **Operating system(s)**: Developed in Linux and platform independent for accessing

• **Programming language**: Java 1.5 and R 2.5.1

• **Other requirements**: Internet Explorer, Firefox or Safari is required to access the website.

## Abbreviations

GEO: Gene Expression Omnibus; MeSH: Medical Subject Headings; SAM: Significance Analysis of Microarray; NCBI: National Center for Biotechnology Information.

## Authors' contributions

YY and KT made substantial contributions to conception and design of GEOGLE as well as in preparing the manuscript. SZ and GD also participated in the construction of GEOGLE and gave important suggestion to the manuscript. JP was involved in the maintaining of the web server. PH and YL participated in the design of the study and preparing of the manuscript. All authors read and approved the final manuscript.

## Supplementary Material

Additional file 1 **GEOGLE – Supplementary Material**. The supplementary materials of GEOGLE for user manual and the description of methods. Click here for file
