# Supplementary material for: GEOGLE: context mining tool for the correlation between gene expression and the phenotypic distinction
Source: BMC Bioinformatics. 2009 Aug 25;10:264. doi: 10.1186/1471-2105-10-264 (PMC2745391; doi:10.1186/1471-2105-10-264)
Supplement: Additional file 1 — GEOGLE – Supplementary Material. The supplementary materials of GEOGLE for user manual and the description of methods. [file 1471-2105-10-264-S1.doc]

# **GEOGLE - Supplementary Material**

Yao Yu^1*^, Kang Tu^1*^, Pei Hao^2§^, Yixue Li^1,2§^

^1^ Bioinformatics Center, Key Lab of Systems Biology, Shanghai Institutes for Biological Sciences, Chinese Academy of Sciences, Shanghai 200031, China,

^2^ SCBIT-Inforsense Joint Lab, Shanghai Center for Bioinformation Technology, 100 Qinzhou Road, Shanghai 200235, China,

Email addresses:

YY: yuyao@scbit.org

KT: ktu@scbit.org

PH: phao@sibs.ac.cn

YL: yxli@sibs.ac.cn

**Outline**

**1. About GEOGLE**

**2. Methods and Materials**

**2.1 Database system**

**2.2 Datasets description**

**2.3 Strategies of miners in GEOGLE**

**2.4 Technical details for calculation**

**2.4.1 Data preprocessing**

**2.4.2 Procedure of SAM**

**2.4.3 Integration of p values**

**3. A step-by-step tutorial**

**3.1 From the very beginning**

**3.2 Miners contained in GEOGLE**

**3.2.1 Vocabulary Miner**

**3.2.2 GDS Miner**

**3.2.3 Pathway Miner**

**3.2.4 Gene Miner**

**3.2.5 GDS BLAST**

**3.3 Output format from GEOGLE**

**3.4 Retrieve results from GEOGLE**

**4. Future plan**

**5. FAQs**

**5.1 What are the data sources of GEOGLE?**

**5.2 Why should I use GEOGLE?**

**5.3 Does GEOGLE have browser preference?**

**5.4 How can I give suggestions and advices?**

**6. About the authors**

**1. A short introduction to GEOGLE**

We developed a state-of-the-art bioinformatics online platform, named GEOGLE, for mining the experimental data from GEO database and constructing the relationships among datasets, genes, pathways and gene biological meaning. We system integrates information from multi sources, such as sigPathway (Tian, Greenberg, Kong, Altschuler, Kohane and Park 2005) for pathway information, and MeSH for biomedical vocabulary. Investigators are able to use multi forms of data for querying, including disease information, gene names, pathway names, GDS ID, and signature lists, to search a large collection of related microarray information. This mining technology may have great value in discovering the linkage between known phenotype and experiment data, as well as retrieving suitable datasets for further research work.

**2. Methods and Materials**

**2.1 Database system**

GEOGLE was built on ‘Omics Explorer’ bioinformatics online service platform, integrating R statistics platform. GEOGLE updates the integrated database frequently.

**2.2 Datasets description**

Currently, GEOGLE collected gene expression datasets from GEO of three species: Human, Mouse and Rat. In total 1005 GDSes from 21 platforms generated with very heterogeneous experimental procedures have been collected in GEOGLE. 6 GPLs in Human contain 351 GDSes, while 9 GPLs in Mouse contain 500 GDSes and 6 GPLs in Rat contain 154 GDSes. The details of these datasets are available from our web site.

Each dataset derived was automatically normalized while missing values were imputed, then was divided into different groups of experimental factors (e.g. tissue, strain, time, dose, etc) according to the description. The following process is to calculate p value of each gene from the comparison of the log2 transformation of their intensities with Significance Analysis of Microarray (SAM) method. Also biological vocabulary information was collected from MeSH. Considering the hierarchical structure of MeSH, we automatically associated the corresponding MeSH terms to all its stored synonyms. Besides, some GEO datasets will be filtered out because of the limited chip numbers, which might be considered not suitable for meta-analysis.

**2.3 Strategies of miners in GEOGLE**

Five miners were included in GEOGLE. They are Vocabulary Miner, GDS Miner, Pathway Miner, Gene Miner, GDS BLAST. We implement them as following.

**Vocabulary Miner**: The inputted words (one or several vocabularies) submitted are mapped to a series of related GDS using MESH dictionary and the description of GDS. P value which describes the relationship between each gene and the group of GDSes is calculated based on the individual p values between each genes and each GDS which have been calculated in advance. Meanwhile, the P value which describes the relationship between each pathway and the group of GDSes is calculated. At last GDSes, genes and pathways are individually sorted based on the p value and given out with the summarized p value and detail p values.

**GDE Miner**: The strategy of this miner is similar with Vocabulary Miner. The list of the group of candidate GDSes is given by user, instead of being obtained by vocabulary searching.

**Pathway Miner**: User submits a name or a part of the name of one pathway at first. Then, the inputted names are mapped to one or several related pathways. After that, a set of genes which related with the pathway are found out. P value which describes the relationship between each GDS and the set of genes is calculated based on the individual p values between each genes and each GDS which had been calculated in advance. In the same time P value which describes the relationship between each vocabulary and the group of GDSes is calculated. At last the GDSes and vocabularies were individually sorted based on the p value and wrote out with the summarized p value and detail p values.

**Gene Miner**: The strategy of this miner is similar with Pathway Miner. The list of the group of candidate genes is given by user, instead of being obtained by pathway vocabulary searching.

**GDS BLAST**: User submits p value list (or ranks) for each gene from a microarray dataset (treatment vs control). To measure the similarity between each dataset in GEO and the user submitted dataset Pearson’s correlation coefficients are measured between the p value list of user submitted dataset and that from the GDSes in GEO. Our strategy to find out whether one GDS or one vocabulary are significantly similar with the submitted dataset than other ones to compare the Pearson’s correlation coefficients of GDS or several GDSes related with one vocabulary, with them of other ones, by one-sided Wilcox rank test. Finally, the GDS and vocabularies are separately sorted based on the p value of Wilcox test and given out.

**2.4 Calculation strategy**

**2.4.1 Data preprocessing**

For a GDS, microarrays were organized in several groups with different treatments. All gene expression datasets were download using R packages (such as ‘GEOquery’). The basic description of the experiment conditions are stored in the meta information of each dataset. By parsing the meta information, expression data from each dataset could be classified into several groups. For example, GDS1304 (title: Cigarette smoking effect on small airway epithelium) contains 11 examples. According to their description, they can be classified into two groups: non-smoker and somker.


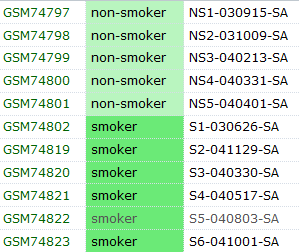


**2.4.2 Procedure of SAM**

Then p value of each gene was calculated from the comparison of the log2 transformation of their intensities with Significance Analysis of Microarray (SAM) method. SAM is a statistical technique for finding significant genes in a set of microarray experiments. It was proposed by Tusher, Tibshirani and Chu. SAM computes a statistic *di* for each gene *i*, measuring the strength of the relationship between gene expression and the response variable. It uses repeated permutations of the data to determine if the expression of any genes are significantly related to the response. The cutoff for significance is determined by a tuning parameter delta, chosen by the user based on the false positive rate. According to the reference about SAM, technical details of the SAM procedure are given below:

The data is *x_ij_* , *i* = 1, 2, . . . *p* genes, *j* = 1, 2, . . . *n* samples, and response data *y_j_* , *j* = 1, 2, . . . *n* (*y_j_* may be a vector).

(1) Compute a statistic


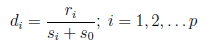


*r_i_* is a score, *s_i_* is a standard deviation, and *s_0_* is an exchangeability factor. Details of these quantities are given later in this note.

(2) Compute order statistics *d_(1)_* < *d_(2)_* · · · < *d_(p)_*

(3) Take B sets of permutations of the response values *y_j_*. For each permutation b compute statistics *d^*b^_i_* and corresponding order statistics *d^*b^ _(1)_* < *d^*b^_(2)_* · · · < *d^*b^ _(p)_*.

(4) From the set of B permutations, estimate the expected order statistics by


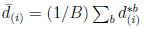
 for *i* = 1, 2, . . . *p*.

(5) For a fixed threshold *E*, starting at the origin, and moving up to the right find the first *i* = *i_1_* such that d_(i)_ − D_(i)_ > *E*. All genes past i1 are called “significant positive”. Similarly, start at origin, move down to the left and find the first *i* = *i_2_* such that *D_(i)_* − *d_(i)_* > *E*. All genes past *i_2_* are called “significant negative”. For each *E* define the upper cut-point cut_up_(*E*) as the smallest *d_i_* among the significant positive genes, and similarly define the lower cut-point cut_low_(*E*).

(6) For a grid of *E* values, compute the total number of significant genes (from the previous step), and the median number of falsely called genes, by computing the median number of values among each of the B sets of *d^*b^_(i)_*, *i* = 1, 2, . . . p, that fall above cut_up_(*E*) or below cut_low_(*E*). Similarly for the 90th percentile of falsely called genes.

(7) Estimate ∏, the proportion of true null (unaffected) genes in the data set.

(8) The median and 90th percentile of the number of falsely called genes from step 6, are multiplied by ∏.

(9) User then picks a *E* and the significant genes are listed.

(10) The False Discovery Rate (FDR) is computed as [median (or 90th percentile) of the number of falsely called genes] divided by [the number of genes called significant].

(Other material for SAM could be found in <http://www-stat.stanford.edu/~tibs/SAM/>)

**2.4.3 Integration of p values**

After that, p values of different genes in each dataset were organized into a vector. Then gene - GDS matrices (named *P_gc_* , *gc* for gene – condition) were generated from a set of p values vectors calculated independently from different GDSes. Each element in *P_gc_* represents a p value which had been prepared before. What we want to know is if these genes in *P_gc_* are perturbed under a group of GDSes, which equal to test if the sub matrix of *P_gc_* of those genes and those GDSes (the sub matrix is named *Pgc_sub*) follow uniform distribution. To perform such a test, *Pgc_sub* were transformed to *Zgc_sub* with quantile function of normal distribution. (See in *Gilchrist, W. (2000). Statistical Modelling with Quantile Functions*.) Then Z score was summarized from *Zgc_sub* with the function:

, *n* for the size of matrix *Zgc_sub*

Our preconception is if these genes were not signatures of a group of GDSes, the *Pgc_sub* would follow uniform distribution range from 0 to 1. If *Pgc_sub* followed uniform distribution, *Zgc_sub* would follow norm distribution. As a result, Z score would also follow norm distribution. A significant small value of Z comparing to normal distribution corresponded to the significantly being perturbed of these genes under these conditions. Using the cumulative distribution function of normal distribution, we calculated a new p value to represent the significance of *Z* score. A very significant p value represents that *Zgc_sub* does not follow norm distribution, thus this gene should be considered as signature of this group of GDSes. With a proper threshold (parameter as ‘alpha’), significant groups of GDSes could be found out given a group of genes, and significant groups of genes could be also found out given a group of GDSes vice versa. The new p value from this process is considered as an index to represent the relationship between the signatures and the whole set of candidate related dataset, by integrating information from individual p values.

Procedures:

Step (1) to (5) were performed to calculated the relationship between candidate signatures and a group of phenotype related GDSes (or expression datasets):

1. Construct the *P_gc_* matrix for collection p values of genes in GDSes according to SAM.
2. *Pgc_sub* were transformed to *Zgc_sub* with quantile function of normal distribution

A quantile function of a probability distribution is the inverse *F*^−1^ of its cumulative distribution function (cdf) *F*. Assuming a continuous and strictly monotonic distribution function,
*F:R* -> (0,1), the quantile function returns the value below which random draws from the given distribution would fall, *p*×100 percent of the time. That is, it returns the value of x such that

The cumulative distribution function (cdf) of the normal distribution is expressed in terms of the density function as follows:

1. Then *Z* score was summarized from *Zgc_sub* with the function:
2. A new p value was calculated to represent the significance of *Z* score using the cumulative distribution function of normal distribution (as mentioned in (2)).
3. Let a parameter (‘alpha’) be the threshold of p values from this test. The p value from this test is considered as the integrated p value from the whole searching task. We could judge whether certain gene should be considered as signature in the group of GDSes by the integrated p value.
4. To judging the relationship between candidate signatures and vocabularies is very similar with the procedure (1) to (5) mentioned. Each vocabulary (terms in MeSH) contains a groups of GDSes mapped in the first engine for datasets collection and signature extraction. The integrated p value for the correlation between signatures and vocabulary is equal to the integrated p value for the correlation between signatures and the GDSes in certain vocabulary.
5. The next step is to evaluate the relationship between pathway and phenotype. We will use a very similar procedure with some modification. This relationship is represented by the correlation between certain pathway and a group of genes (signatures). We constructed a pathway - gene matrices (named *P_pg_* , *pg* for pathway - gene). Each element in this matrix is the integrated p value of gene in a group of GDSes (this group is determined according pervious GDS searching). Then the procedure of (1) to (5) was performed, using *P_pg_* taking the place of *P_gc_*. The new integrated p value calculated was considered to be the estimate of the significance of the pathway in the searching task.

**3. A step-by-step tutorial**

**3.1 From the very beginning**

The rapid growth of high-throughput microarray technology provides a huge amount of experimental data for advanced research associating gene expression signatures with biological phenotypes. For example, the application of microarrays to identify gene expression signatures of human diseases has been widely accepted. To address the problems of analyzing often limited samples in biological experiments and heterogeneous gene expression datasets from different sources, methods for large-scale meta-analysis of microarray data have been developed, such as Connectivity Map.

Before using GEOGLE, users should also have some basic knowledge of some resources. GEOGLE is an online querying platform for Gene Expression Omnibus (GEO) and associated MeSH and SigPathway entries. The Gene Expression Omnibus (GEO) (Edgar, Domrachev and Lash 2002), curated by the National Center for Biotechnology Information (NCBI), is designed in response to this demand for a public warehouse for the submission, storage and retrieval of the high-throughput gene expression and genomic hybridization experiments. Medical Subject Headings (MeSH) (Lowe and Barnett 1994) is one of the best resources for biomedical vocabularies. sigPathway is an R package that performs pathway (gene set) analysis on microarray data.

From the very beginning, users have to **login** in Omics Explorer. The **demo** account is available to all visitors here. This part is important of checking the running stats of initialized tasks and retrieving searching results. Generally, the login process will be finished *automatically* when a visitor without personal account in Omics Explorer accesses to our website. In case the automatical login system does not work, users have to use the following form from the left of the window to access into GEOGLE manually.


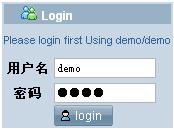


**3.2 Miners contained in GEOGLE**

Then users should choose the miner(s) to use from the list. Five major mining tools have been provided with a user-friendly interface: Vocabulary Mining, GDS Mining, Pathway Mining, Gene Mining and Blast Mining.


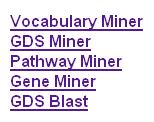


**3.2.1 Vocabulary Miner**

Mining out common signature genes and pathways based on biomedical vocabularies. User should submit some key words of their interest. This miner will searching the corresponding datasets and summarize the signature genes from these datasets as well as their biological annotation and pathways.

(1) Choose species: human, mouse or rat.

(2) A disease related vocabulary should be given, for instance 'smoking'.

(3) Submit the data by clicking 'GDS' button, the results will be returned containing five major fields: GDS, GPL, LIB, TITLE and DESCRIPTION.

(4) Submit the data by clicking 'MeSH' button, the results will be returned containing three major fields: Term_ID, Term_Name and TITLE_DESCRIPTION_GDS.

(5) Some optional parameters can be set by users, for instance set FALSE for 'listMeshGDS.only' as default will make the miner search all related information including 'Gene' and 'Pathway' according to the query, which is believed taking a longer time. A fast search for only MESH words and GDS IDs could be done with TRUE set for the parameter, no results will be returned by clicking 'Gene' or 'Pathway' button.

(6) For 'alpha': this one is set for the threshold of statistical value for judging significantly outstanding results. Please find the processing of calculation in method part.

Illustration:


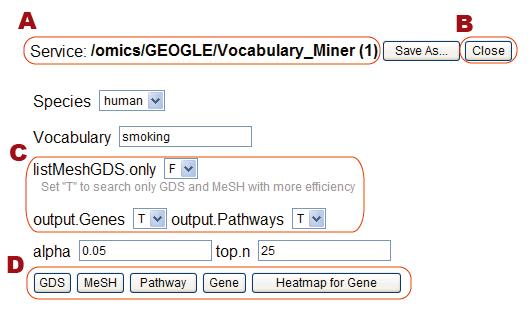


**A**: The title for the miner

**B**: A button for closing this miner after using

**C**: Some special optional parameters

for instance setting FALSE for '**listMeshGDS.only**' as default will make the miner search all related information including '**Gene**' and '**Pathway**' according to the query, which is believed taking a longer time. A fast search for only MESH words and GDS IDs could be done with TRUE set for the parameter, no results will be returned by clicking '**Gene**' or '**Pathway**' button. '**output.Gene**' and ' **output.Pathway**' let the users to choose if they want to retrieval the gene and pathway information or not when FALSE is set for '**listMeshGDS.only**'.

**D**: The output buttons. Please use any of them to get the results interested

**3.2.2 GDS Miner**

Retrieving signature genes and extracting related pathway information based on given datasets (usually GDS IDs). This miner is similar with the first one. However the GDSes should be directly given by the user instead of being found by search key words. Also this miner can be helpful in searching for the annotation information of datasets with GDS IDs.

(1) Choose species: human, mouse or rat;

(2) A list of GDS IDs should be typed in the following text field.;

(3) Submit the data by clicking 'GDS' button, the results will be returned containing five major fields: GDS, GPL, LIB, TITLE and DESCRIPTION;

(4) Submit the data by clicking 'Pathway' button, the results will be returned containing two major fields: Pathwayand P_value;

(5) Submit the data by clicking 'Gene' button, the results will be returned containing: GeneID, Symbol, P_value and P_values in different GDSes

(6) For 'alpha': the one is set for the threshold of statistical value for judging significantly outstanding results.

Illustation:


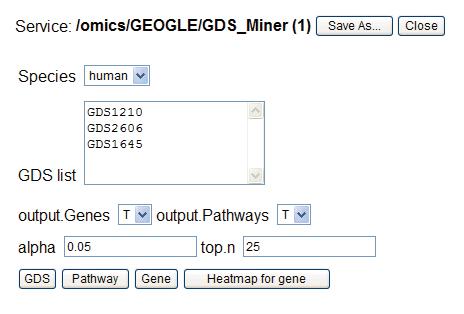


**3.2.3 Pathway Miner**

Rretrieving related datasets and biomedical vocabularies based on submitted pathways. Users may submit certain pathway name or partial name. This miner will help to obtain those genes within the pathway. Then the GDSes in which these genes are considered as signature will be return as well as their annotation.

(1) Choose species: human, mouse or rat.

(2) A pathway name (or similar words) should be given, for instance 'regulation of tumor'

(3) Submit the data by clicking 'Pathway' button, the results will be returned containing two major fields: pathway Source for GO IDes and their Name

(4) Set FALSE for 'listPathway.only' will make the miner all related information according to the query, which is believed taking a longer time. This results will not be returned by set TRUE for this parameter, which is similar to 'Vocabulary Miner'.

(5) For 'alpha': the one is set for the threshold of statistical value for judging significantly outstanding results.

Illustration:


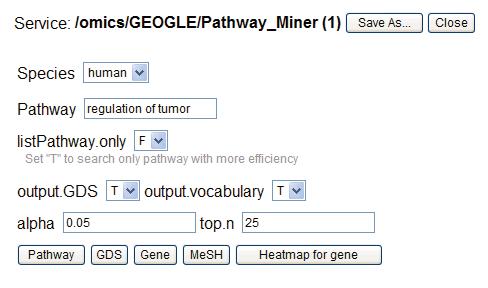


**3.2.4 Gene Miner**

Searching with a given gene list which might be considered as signatures in several GDSes. User can provide genes of their interest directly. Then the miner will search for the corresponding GDSes in which these genes are considered to be signatures. This process is quite similar with Pathway Miner.

(1) Choose species: human, mouse or rat.

(2) A list of Genes should be typed in the following text field.

(3) Submit the data by clicking 'GDS' button, the results will be returned containing six major fields: GDS, GPL, LIB, TITLE, DESCRIPTION and P value.

(4) Submit the data by clicking 'Gene' button, the results will be returned containing: GeneID, Symbol, P_value and related GDSes

(5) Submit the data by clicking 'MeSH' button, the results will be returned containing three major fields: Term_ID, Term_Name and TITLE_DESCRIPTION_GDS

(6) 'alpha': the one is set for the threshold of statistical value for judging significantly outstanding results.

Illustration:


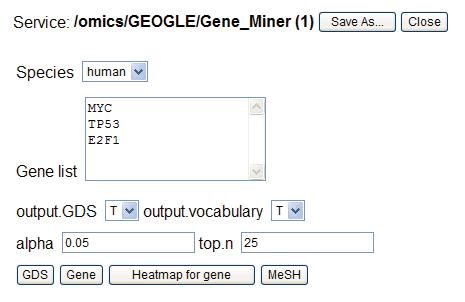


**3.2.5 GDS BLAST**

Searching datasets similar to submitted microarray datasets (namely a list of signatures) and then summarizing associated biomedical vocabularies. Users may submit a list of signature according to their own defination. What this miner will perform is to searching for similar dataset using the same (or almost same) group of genes as signatures.

Firstly, user should choose the species and the platform for quering.

The list of signature should be given in the form of two columns:probe_ID and rank value (for example FDR values); and these two columns are splited by comma.

Using the output.GDS and output.vocabulary to filter the result if users do not need some part of the result and want to have a faster querying.

Illustration:


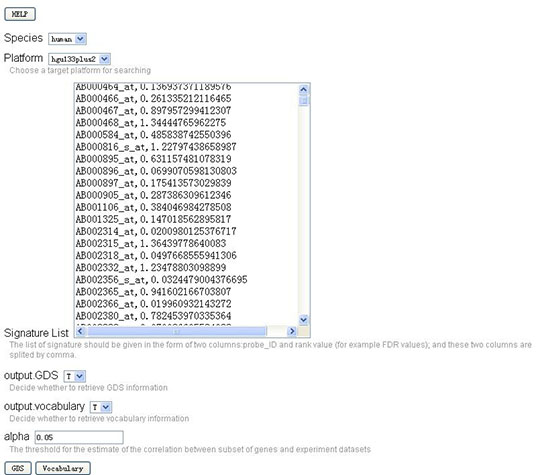


**3.3 Output format from GEOGLE**

After submitting the query genes by clicking those buttons, a basic result page will be returned. Generally speaking, table format results can be contained in the result. The detail explanations of outputs from those five workflows can be found from the ‘**HELP’** in the execution page of each miner.

Output Explanation：

1. GDS table contains six columns:
   1. GDS_ID: gene expression dataset identify from GEO
   2. GPL_ID: gene expression platform identify from GEO
   3. LIB: platform names
   4. TTILE: GDS title
   5. DESCRIPTION: GDS description
   6. GDS_link: web link for accessing GDS (please choose HTML renderer to use the hyperlink)
2. MeSH table contains three columns
   1. Term_ID: term IDs in MeSH
   2. Term_Name: MeSH terms’ names
   3. TITLE_DESCRIPTION_GDS: mapped GDSes according to query
3. Gene table contains over five columns
   1. GENEID: EntrezGene ID
   2. Symbols: gene names
   3. P_value: The composite p value represents the significance of the correlation between gene expression and phenotypic distinction (users’ query).
   4. Several columns of the P_values in different GDSes: The individual p value followed represents whether a gene should be considered as a signature from individual expression dataset. (A NA value in the P_value field means the gene is missing in the corresponding GDS)
   5. GENE_link: web link for accessing gene (please choose HTML renderer to use the hyperlink)
4. Pathway table contains over five columns
   1. PATHWAY: pathway names
   2. P_value: the p value represents the significance of the correlation between g PATHWAY and phenotypic distinction (users’ query).
5. Heatmap for Gene: A heatmap figure for the gene table result (genes by GDSes) can be downloaded from GEOGLE in PDF format.
6. Histplot for Gene: Histplot for the distribution of p_values representing the relationship between gene expression and phenotypic distinction
7. Histplot for pathway: Histplot for the distribution of p_values representing the relationship between pathway and phenotypic distinction

For the result page, there are several special functions. Users can download a table format result in different file formats, including CSV (Excel), TSV, XML(WebRowSet XSD), and SFS(Spotfire). If there are too many records contained in the table, only the first 30 lines will be displayed in the default result page. The number of records to be displayed can be changed by setting a number for the ‘sampling’ field and clicking the ‘update’ button.


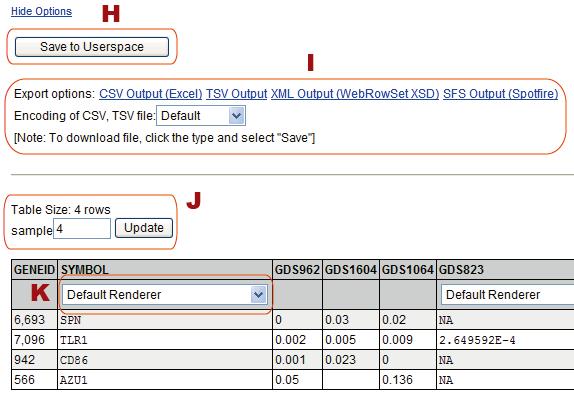


**H**: The results can be saved into users' space for furthre usage.

**I**: The output can be exported in various formats.

**J**: Users can set a number to sample partial records from the whole results

**K**: The renderer of records in the table can be changed.

The **figure** results from GEOGLE are given either in the web pages or from PDF files.

**Use HTML Render**

We provide a link for genes and GDSes in the last column from the result table, like:


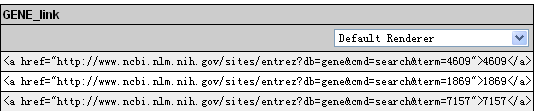


As default renderer users can view the link detail of each gene. By change the renderer type, the users are free to choose to use hyperlink for these genes (or GDSes).


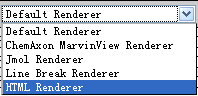


Using HTML Renderer, the users will get the results with links, which looks like:


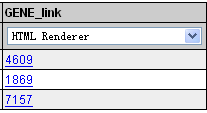


**3.4 Retrieve results from GEOGLE**

The GEOGLE system will record previously conducted task and save the results on the sever. Thus users can retrieve these results from GEOGLE’s website. After automatically login into GEOGEL, users can click the ‘task’ button on the menu bar to view all recorded result:


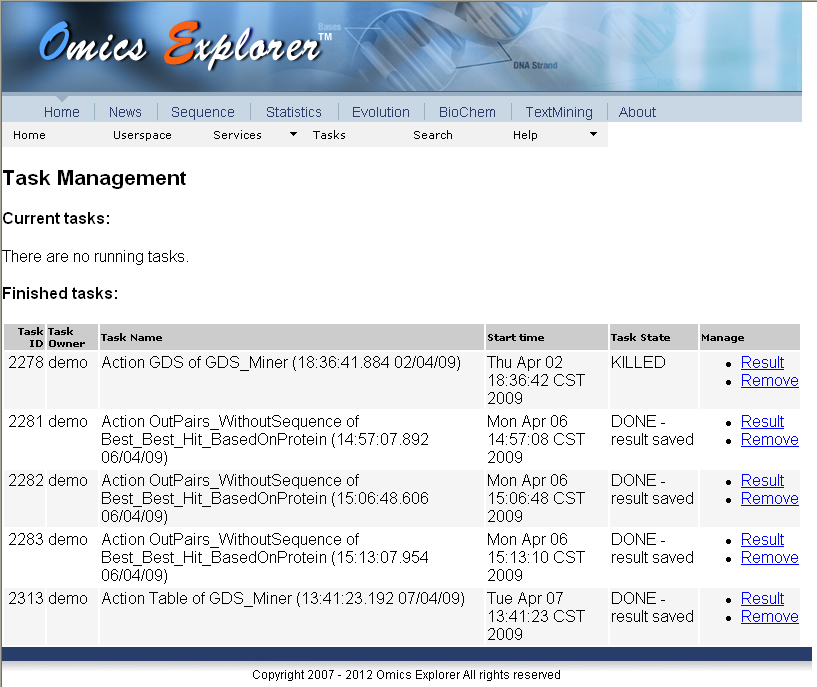


(Pay attention: the language depends on system language)

Click the ‘Result’ link will lead the users to retrieve their previous task results.

Click the ‘Remove’ link will lead the users to remove their previous task results.

Two points have to be noted:

1. Users have to remember the task ID from the first column or remember the time they started the task.

2. These results are only temporarily saved on the sever; the system will only keep these for a few days without any additional requirements.

**4. Future plan**

Further steps in the development of GEOGLE would focus on the integration of high-throughput gene expression databases other than GEO. One of the improvements of GEOGLE that is in-progress is large scale gene and disease information mining effort from reference databases and integrating this information with existing signature data. The reference mining results are believed to be able to prove the reliability of the relationships between signatures and diseases discovered by GEOGLE. Moreover, since GEOGLE provides a potential network of diseases, genes and pathways, more analysis work focusing on this will be considered in future.

**5. Frequently Asked Questions**

**5.1 What are the data sources of GEOGLE?**

GEOGLE uses an automatic integrating system to collecting information for GEO, MeSH and SigPathway, as well as constructing the relationships among them.

**5.2 Why should I use GEOGLE?**

Several tools and strategies for operating the GEO database have been developed to enable comparisons of microarray data across experimental platforms, different laboratories and multiple species. However most of these tools for retrieving data from the GEO repository paid little attention to mining further information about the gene expression signatures, such as the biological meaning and functions of genes or integrating the related pathway information in the biological processes. Medical Subject Headings (MeSH) is one of the best resources for biomedical vocabularies, but no integrated tool for mining signature-related information from MeSH is currently available. GEOGLE is designed to provide a final solution for these problems.

**5.3 Does GEOGLE have browser preference?**

GEOGLE was developed and tested under IE6.0 and firefox 2.0. If your browser has problems with GEOGLE, please contact us: yyu01@sibs.ac.cn

**5.4 How can I give suggestions and advices?**

Our contact email is yyu01@sibs.ac.cn, if you have any question or suggestion, please don't hesitate to contact us.

**6. About the author**

Yao Yu, Email:yyu01@sibs.ac.cn

Shanghai Institutes for Biological Sciences

Kang Tu, Email:ktu@sibs.ac.cn

Shanghai Institutes for Biological Sciences

Pei Hao, Email:phao@sibs.ac.cn

Shanghai Institutes for Biological Sciences

Shanghai Centre for Biotechnology

Yixue Li, Email:yxli@sibs.ac.cn

Shanghai Institutes for Biological Sciences

Shanghai Centre for Biotechnology

Bioinformatics center, Key Lab of Systems Biology, SIBS, CAS. 2008. All Rights Reserved.
